# Point-Based Prediction Model for Bladder Cancer Risk in Diabetes: A Random Survival Forest-Guided Approach

**DOI:** 10.3390/jcm14010004

**Published:** 2024-12-24

**Authors:** Sarah Tsz Yui Yau, Chi Tim Hung, Eman Yee Man Leung, Ka Chun Chong, Albert Lee, Eng Kiong Yeoh

**Affiliations:** JC School of Public Health and Primary Care, The Chinese University of Hong Kong, Hong Kong, China

**Keywords:** diabetes, bladder cancer, risk prediction, survival analysis, random forest

## Abstract

**Background:** Previous epidemiological studies have shown that diabetes is associated with an increased risk of several cancers, including bladder cancer. However, prediction models for bladder cancer among diabetes patients remain scarce. This study aims to develop a scoring system for bladder cancer risk prediction among diabetes patients who receive routine care in general outpatient clinics using a machine learning-guided approach. **Methods:** A territory-wide retrospective cohort study was conducted using electronic health records of Hong Kong. Patients who received diabetes care in public general outpatient clinics between 2010 and 2019 without a history of malignancy were identified and followed up until December 2019. To develop a scoring system for bladder cancer risk prediction, random survival forest was employed to guide variable selection, and Cox regression was subsequently applied for weight assignment. **Results:** Of the 382,770 patients identified, 644 patients developed bladder cancer during follow-up (median: 6.2 years). The incidence rate was 0.29 per 1000 person-years. In the final time-to-event scoring system, age, serum creatinine, sex, and smoking were included as predictors. Serum creatinine ≥94 µmol/L appeared to be associated with an increased risk of developing bladder cancer. The 2-year and 5-year AUCs on test set were 0.88 (95%CI: 0.84–0.92) and 0.86 (95%CI: 0.80–0.92) respectively. **Conclusions:** Renal dysfunction could be a potential predictor of bladder cancer among diabetes patients. The proposed scoring system could be potentially useful for providing individualized risk prediction among diabetes patients.

## 1. Introduction

Globally, bladder cancer ranks ninth in cancer incidence, with over 600,000 newly diagnosed cases each year [1]. Previous research has shown that bladder cancer is more common among patients with diabetes than the general population [2]. However, apart from tobacco smoking and occupational/environmental exposures to carcinogens, few risk factors for bladder cancer have been identified with strong evidence [3].

Existing prediction models for bladder cancer tend to focus on symptoms of bladder diseases [4]. Most of these models incorporate the presence of visible or non-visible hematuria as predictors, and some of them also include clinical signs such as abdominal pain, loss of appetite, or weight loss [4]. While smoking is a common predictor across most models [4], other modifiable factors are less commonly incorporated, except among a few models developed in primary care. For example, in a model designed to predict multiple cancers [5,6], alcohol use, body mass index (BMI), and diabetes have also been included. Nevertheless, in another model specific to renal tract cancer prediction in primary care [7], neither alcohol use, BMI, diabetes, or treated hypertension examined in the set of candidate predictors has been included in the final model.

Several scoring systems for bladder cancer have been developed to facilitate risk stratification among patients with the presentation of hematuria, where age, sex, smoking status, and hematuria (visible vs non-visible) are commonly included as predictors [8,9]. However, scoring systems have rarely been developed among the general population [5,6,7], or under a setting where hematuria cannot be routinely tested or systematically coded. Furthermore, bladder cancer is more commonly diagnosed in patients with diabetes than the general population [2]. Nevertheless, prediction models for bladder cancer among patients with diabetes remain rare, and prediction models among diabetes patients in the absence of bladder disease symptoms have not been developed.

While logistic regression or Cox regression has been applied to develop risk prediction models for bladder cancer by convention [4], several studies [10,11] have attempted to apply different machine-learning algorithms, such as support vector machine, naïve bayes, and tree-structured algorithms (including decision tree, random forest, and gradient boosting), to identify factors associated with bladder cancer and compare model performance across different algorithms. These studies showed that random forest demonstrated good performance when compared to other algorithms. Nevertheless, variable importance in these studies was reported in purity metrics (information gain and Gini index) and this may not necessarily provide a quantifiable magnitude of risk for interpretation to inform clinical practice or health policy making. Moreover, these studies examined binary bladder cancer outcome and did not incorporate the time component in outcome measures.

Recently, a machine learning-integrated approach [12] to develop clinical scoring systems for time-to-event outcome has been proposed, where random survival forest is first employed to guide variable selection, and Cox regression is subsequently applied to assign weights to each variable in the scoring model. Clinical expert knowledge is taken into account in model fine-tuning. The advantages of this proposed approach are as follows: (i) use of random survival forest provides a less biased approach in selecting less established risk factors for model building and reduces variance when compared to individual trees; (ii) Cox regression remains the most widely accepted approach in deriving prediction models and scoring systems for time-to-event health outcomes; (iii) scoring systems could be more useful for risk stratification and clinical application than prediction models alone; and (iv) clinical expert knowledge is incorporated in model development to ensure clinical relevance.

To address the research gap on the paucity of prediction models or scoring systems for bladder cancer among diabetes patients, this study seeks to develop a random survival forest-guided scoring system to predict the risk of bladder cancer among diabetes patients who receive routine care in general outpatient clinics.

## 2. Methods

### 2.1. Study Design and Study Population

A territory-wide retrospective cohort study was performed using electronic health records of Hong Kong. The Hospital Authority (HA) is a statutory body providing public healthcare services and maintains a centralized clinical data repository that stores information on patients’ demographics, disease diagnoses, prescription records, laboratory measurements, inpatient admissions, and outpatient attendances. Disease diagnoses were coded according to the International Classification of Disease 9th or 10th revision (ICD-9 or ICD-10), or the International Classification of Primary Care 2nd edition (ICPC-2). Data was accessed via HA Data Collaboration Lab.

### 2.2. Patients

Patients who received a diagnosis of type 2 diabetes and underwent a first diabetes mellitus complication screening (DMCS) assessment in general outpatient clinics between 2010 and 2019 were initially included. Type 2 diabetes was defined as a clinical diagnosis confirmed by clinicians, with two abnormal test results of plasma glucose and presentation of clinical symptoms. Index date was defined as date of the initial DMCS assessment. Those who (i) received a diagnosis of diabetes below 18 years old; (ii) had a history of malignancy prior to the baseline assessment; (iii) developed cancer at sites other than bladder; or (iv) had a follow-up period of less than six months were excluded. End date of follow-up was defined as the earliest occurrence time of a bladder cancer diagnosis, death, or study end (31 December 2019).

### 2.3. Outcome

The outcome of interest was diagnosis of bladder cancer (ICD-9: 188; ICD-10: C67) during follow-up. While the diabetes cohort received routine diabetes care in general outpatient clinics, the diagnosis of bladder cancer was extracted from inpatient hospital records. Bladder cancer is not routinely tested in clinics. Nevertheless, patients with suspected bladder cancer could be referred to specialists for further testing if necessary. Patients are flagged as suspected bladder cancer when they present with blood in their urine, pain during urination, or other urinary symptoms. For bladder cancer diagnosis, a cystoscopy procedure was first performed by urologists for examination. During the procedure, biopsy samples were taken for further examination by pathologists to provide a diagnosis based on the tissue samples.

### 2.4. Input Variables

Information on input variables was ascertained at the time of baseline assessment. Variables included demographics (age and sex), duration of diabetes, behavioral factors (smoking and alcohol use), medical history, medication use, anthropometric measurements (BMI and waist-to-hip ratio), and laboratory measurements. Medical history included cardiovascular diseases (ischemic heart disease, cerebrovascular disease and heart failure), hypertension, respiratory diseases (chronic obstructive pulmonary disease and pneumonia), chronic kidney disease, liver cirrhosis, and family history of diabetes. Medication use included commonly used anti-diabetic drugs (metformin, sulfonylurea, insulin and dipeptidyl peptidase-4 inhibitors), aspirin, non-steroidal anti-inflammatory drugs, anti-coagulants, anti-platelets, anti-hypertensive drugs, and statins. Medication use was defined as whether patients had received a drug at the time of baseline assessment. Laboratory measurements included serum creatinine, HbA_1c_, fasting glucose, low-density lipoprotein cholesterol, high-density lipoprotein cholesterol, and triglycerides. For laboratory measurements, the most recent results prior to the assessment were taken.

### 2.5. Data Analysis

For model building, patients who developed bladder cancer (*n* = 644) and a subset of patients who did not develop bladder cancer (*n* = 19,320) were selected at random in a 1:30 ratio, where the choice of ratio takes into account the optimal class distribution and statistical power. Patients were randomly split into training, validation, and test sets in a default ratio of 7:1:2 [12]. Conventionally, 70% of the data is used for training the model. For the rest of the data, 10% were used for selecting and fine-tuning parameters in the trained model, and 20% were reserved as unseen test set to evaluate performance of the final model. The set of input variables was first entered into a random survival forest model and ranked by their importance, where variable importance in random forest represents the average results of a set of decorrelated decision trees. Variables were selected for the final scoring model by considering model performance improvement and model parsimony, and then assigned weights by applying Cox regression, where the weights were adjusted with reference to all variables in the scoring model. For continuous variables, the cutoff points for setting levels for score assignment were determined by default quantiles [12]. Fine-tuning was performed based on clinical expert knowledge before model finalization. The number of trees in random forest was set as 30. The bladder cancer-free survival probability during follow-up was examined using Kaplan–Meier method. Model performance was evaluated on test set and the full cohort using Harrell’s concordance (C-) index and area under the curve (AUC) as metrics. Data analyses were conducted using R software (version 4.2.3; R Foundation for Statistical Computing, Vienna, Austria).

## 3. Results

A total of 382,770 diabetes patients were identified. During a median follow-up of 6.2 years, 644 patients developed bladder cancer. Patients who subsequently developed bladder cancer tended to be older (71.21 vs 62.33 years, *p* < 0.001), male (77.48 vs 50.64%, *p* < 0.001), ever smoker (53.73 vs 29.72%, *p* < 0.001), and have a higher serum creatinine (98.10 vs 81.53 µmol/L, *p* < 0.001) (Table 1).

### 3.1. Final Scoring System

Age, serum creatinine, sex, and smoking were identified among the top six important variables in the random survival forest model. Commonly used anti-diabetic drugs among the diabetes cohort (metformin, sulfonylurea, insulin, and dipeptidyl peptidase-4 inhibitors) were not ranked among the top fifteen important variables. When each variable was sequentially added to a Cox regression model according to variable importance in the tree model, the addition of these four variables led to a marginal increase in model performance.

These four variables were included in the final Cox regression model and assigned weights based on their beta coefficients. Age, serum creatinine, sex, and smoking were assigned up to 62, 12, 16, and 9 points respectively (Table 2).

Patients started to have a noticeably elevated risk of bladder cancer after reaching the age of 53 years. On the other hand, serum creatinine demonstrated a J-shaped relationship with the risk of bladder cancer. Compared to a level between 51 to 60 µmol/L, serum creatinine below 51 µmol/L contributed to a small increased risk of bladder cancer, and the risk also started to rise from 61 µmol/L and reached to the greatest risk at the level of 126 µmol/L or above. However, only serum creatinine at a level of 94 µmol/L or above demonstrated a comparable risk level as other risk factors in the model. In addition, male sex and smoking were important factors contributing to an increased risk of bladder cancer (Table 2).

### 3.2. Bladder Cancer-Free Survival During Follow-Up

**Figure 1:**
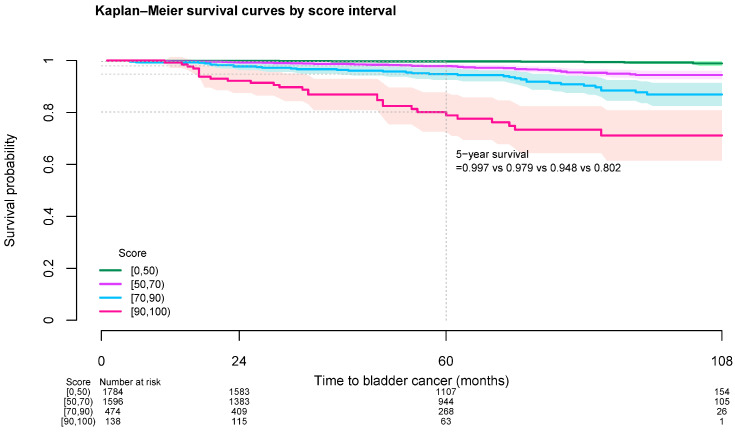
Kaplan-Meier bladder cancer-free survival curves among diabetes patients on test set by risk score.

At five years, the bladder cancer-free survival probability for patients on test set with score 0 to 49, 50 to 69, 70 to 89, and 90 to 99 were 0.997, 0.979, 0.948, and 0.802 respectively. At 7 years, the corresponding survival probability dropped to 0.996, 0.955, 0.908, and 0.734 (Figure 1; Table 3). The proportions of patients who developed bladder cancer during follow-up at each 10-score interval on test set and the entire cohort are shown in Supplementary Table S1 and Table 4 respectively. Among the entire cohort, the proportion of patients with highest score interval (90 to 99) who developed bladder cancer during follow-up was 0.78% (Table 4).

### 3.3. Model Performance

The final scoring system on test set attained a C-index of 0.81 (95% confidence interval [CI]: 0.77–0.84). The 2-year and 5-year AUCs were 0.88 (95%CI: 0.84–0.92) and 0.86 (95%CI: 0.80–0.92) respectively. When the final scoring system was applied to the entire cohort, the C-index was 0.78 (95%CI: 0.77–0.80), indicating acceptable performance [13].

## 4. Discussion

The current study incorporates aggregate information from the multidimensional interactions among covariates in individual survival tress of a random survival forest to guide variable selection, and collapses information into a simple time-to-event risk score for bladder cancer prediction among diabetes patients who receive routine care in primary care clinics. This study demonstrated that apart from tobacco smoking, renal dysfunction could be a potential predictor of bladder cancer among diabetes patients in the presence or absence of bladder cancer symptoms.

The proposed scoring system provides prediction for bladder cancer risk among diabetes patients and demonstrates a comparable model performance with existing scoring systems for symptomatic individuals with hematuria [8,9]. In addition to traditional predictors (namely age, sex, and smoking) [4], serum creatinine was also added as a predictor in the model. Moreover, while most models applied logistic regression for binary outcome in model development [4], the proposed scoring system incorporated time component into the time-to-event outcome. In terms of model performance, the proposed scoring system achieved a comparable overall C-index of 0.805, compared to AUCs ranging from 0.804 to 0.835 in existing scoring systems for symptomatic individuals [8,9].

In the proposed scoring system, serum creatinine exhibited a J-shaped relationship with the risk of bladder cancer, where a slightly elevated risk for the range below 51 µmol/L and a gradual increased risk from 61 µmol/L onwards was observed. While it remains less clear whether a lower limit for optimal serum creatinine level exists [14], a low level of serum creatinine is associated with compromised immune function, reduced muscle mass, and potentially adverse health outcomes [14,15,16]. On the other hand, a high level of serum creatinine is associated with renal dysfunction and potentially elevated risk of bladder cancer. Recent research has shown that renal dysfunction is associated with an increased risk of renal tract cancers [17,18,19], and renal disease treatment could also be associated with bladder cancer [20]. One possible mechanism linking renal dysfunction to bladder cancer is accumulation of exogenous toxins (such as analgesic nephropathy) [21] or increased concentration of uremic toxins [22] (due to reduced urine output) along the urinary tract.

Findings of the study imply that overall, non-smokers aged below 53 years appear to have a low risk of bladder cancer. While smoking remains a strong risk factor for bladder cancer after age and sex, serum creatinine reaching a level of 94 µmol/L or above could also be an indicator of elevated bladder cancer risk. Furthermore, the present study suggests the potential association between renal dysfunction and renal tract cancers [19]. Future research is needed to examine whether improved renal function would potentially lower the risk of bladder cancer among diabetes patients. In addition, given the higher risk of bladder cancer for patients with diabetes, further studies are warranted to explore whether high-risk diabetes patients would require closer monitoring from doctors during their routine visits to primary care clinics for diabetes management or would be recommended for hematuria testing at an initial assessment or at regular intervals.

In comparison with other renal markers, estimated glomerular filtration rate (eGFR) incorporates information on age, sex, and serum creatinine level. The proposed scoring system in the present study includes age, sex, serum creatinine, and smoking as predictors. While serum creatinine is directly measured from blood samples, eGFR is an estimated renal marker. Also, the proposed scoring system has additionally incorporated smoking as predictor, in addition to accounting for age and sex differences.

There are several limitations of the current study. First, occupational exposures to carcinogens are established risk factors for bladder cancer [3]. However, occupational information was not ascertained during routine diabetes care and hence not available in this study. Systematic collection on occupational information may provide an additional candidate predictor for scoring system development and potential model improvement. Second, clinical symptoms such as hematuria may not be systematically coded or routinely tested in primary care, hence hematuria was not incorporated in the proposed scoring system. Nevertheless, the proposed scoring system may provide risk stratification among diabetes patients, and potentially help guide clinical decision on referral for further hematuria testing. Third, dosage and duration of medication use was not evaluated in this study. Fourth, the current study did not have access to external validation. However, the unseen test data was used to evaluate model performance. Lastly, generalizability of the study could be restricted to Asian diabetes population. Future research is warranted to explore other potential predictors of bladder cancer risk (such as inflammatory markers [23]), and generalizability in other populations.

## 5. Conclusions

The present study demonstrated that apart from smoking, renal dysfunction could be a potential predictor of bladder cancer among diabetes patients. This study developed a time-to-event scoring system for bladder cancer risk prediction among diabetes patients regardless of symptoms. In the final scoring system, age, serum creatinine, sex, and smoking were included as predictors. The proposed scoring system could be potentially useful for individualized bladder cancer risk prediction among diabetes patients who receive routine care in primary care clinics.

## Figures and Tables

**Table 1:** Baseline characteristics of diabetes patients by subsequent cancer status.

	Bladder Cancer		No Cancer _random subset_			
Characteristics	(*n* = 644)		(*n* = 19,320)		χ^2^/*t*	*p*
Demographics						
Male, *n* (%)	499	(77.48%)	9783	(50.64%)	179.85	<0.001
Age at assessment in year, mean ± SD	71.21	±9.56	62.33	±11.65	23.01	<0.001
Duration of diabetes in year, median (IQR)	6	(2–12)	3	(1–9)		
Behaviors						
Current or former smoker, *n* (%)	346	(53.73%)	5741	(29.72%)	169.54	<0.001
Current or former drinker, *n* (%)	252	(39.13%)	5658	(29.29%)	28.98	<0.001
Medical history						
Cardiovascular diseases						
Ischemic heart disease, *n* (%)	88	(13.66%)	1340	(6.94%)	42.49	<0.001
Cerebrovascular disease, *n* (%)	58	(9.01%)	1185	(6.13%)	8.81	0.003
Heart failure, *n* (%)	26	(4.04%)	352	(1.82%)	16.47	<0.001
Hypertension, *n* (%)	588	(91.30%)	16,489	(85.35%)	17.88	<0.001
Respiratory diseases						
Chronic obstructive pulmonary disease, *n* (%)	13	(2.02%)	120	(0.62%)	18.39	<0.001
Pneumonia, *n* (%)	38	(5.90%)	580	(3.00%)	17.46	<0.001
Chronic kidney disease, *n* (%)	76	(11.80%)	3026	(15.66%)	7.08	0.008
Liver cirrhosis, *n* (%)	9	(1.40%)	376	(1.95%)	0.99	0.319
Family history of diabetes, *n* (%)	249	(38.66%)	9098	(47.09%)	17.77	<0.001
Medication use						
Anti-diabetic drugs						
Metformin, *n* (%)	362	(56.21%)	7812	(40.43%)	64.15	<0.001
Sulfonylurea, *n* (%)	263	(40.84%)	5274	(27.30%)	57.01	<0.001
Insulin, *n* (%)	55	(8.54%)	1244	(6.44%)	4.52	0.033
Dipeptidyl peptidase-4 inhibitors, *n* (%)	21	(3.26%)	786	(4.07%)	1.05	0.306
Sodium-glucose cotransporter-2 inhibitors, *n* (%)	0	(0%)	59	(0.31%)		
Glucagon-like peptide-1 receptor agonists, *n* (%)	0	(0%)	8	(0.04%)		
Glucosidase inhibitor, *n* (%)	4	(0.62%)	83	(0.43%)		
Glitazone, *n* (%)	3	(0.47%)	71	(0.37%)		
Meglitinide, *n* (%)	0	(0%)	5	(0.03%)		
Any of the above, *n* (%)	467	(72.52%)	10,203	(52.81%)	97.26	<0.001
Aspirin, *n* (%)	207	(32.14%)	4016	(20.79%)	48.19	<0.001
Non-steroidal anti-inflammatory drugs, *n* (%)	325	(50.47%)	10,522	(54.46%)	4.01	0.045
Anti-coagulants, *n* (%)	48	(7.45%)	923	(4.78%)	9.64	0.002
Anti-platelets, *n* (%)	37	(5.75%)	1404	(7.27%)	2.16	0.142
Anti-hypertensive drugs, *n* (%)	496	(77.02%)	13,293	(68.80%)	19.68	<0.001
Statins, *n* (%)	327	(50.78%)	9524	(49.30%)	0.55	0.460
Anthropometric measurements						
Body mass index in kg/m ^2^, mean ± SD	25.34	±3.49	26.07	±4.23	5.18	<0.001
Waist-to-hip ratio, mean ± SD	0.96	±0.06	0.94	±0.06	8.32	<0.001
Laboratory measurements						
Serum creatinine in µmol/L, mean ± SD	98.10	±48.47	81.53	±40.40	8.58	<0.001
HbA_1c_ in %, mean ± SD	7.30	±1.33	7.37	±1.46	1.31	0.190
Fasting glucose in mmol/L, mean ± SD	7.33	±1.92	7.62	±2.25	3.75	<0.001
Low-density lipoprotein cholesterol in mmol/L, mean ± SD	2.61	±0.75	2.67	±0.82	1.99	0.047
High-density lipoprotein cholesterol in mmol/L, mean ± SD	1.23	±0.33	1.27	±0.33	3.03	0.002
Triglycerides in mmol/L, mean ± SD	1.49	±0.92	1.63	±1.26	3.75	<0.001

Comparisons of differences in proportion for categorical variables and mean for continuous variables were performed using chi-squared test and *t*-test respectively.

**Table 2:** Final scoring system for bladder cancer risk prediction among diabetes patients.

Variable	Value	Point
Age, years	Less than 43	0
	43 to 52	3
	53 to 72	38
	73 to 81	56
	82 or above	62
Serum creatinine, µmol/L	Less than 51	3
	51 to 60	0
	61 to 93	3
	94 to 125	9
	126 or above	12
Sex	Female	0
	Male	16
Smoking	Never smoker	0
	Ever smoker	9

**Table 3:** Bladder cancer-free survival probability on test set at different follow-up time points by score interval.

	Score Interval			
Time	0 to 49	50 to 69	70 to 89	90 to 99
t = 2 years	0.999	0.993	0.977	0.922
t = 5 years	0.997	0.979	0.948	0.802
t = 7 years	0.996	0.955	0.908	0.734

**Table 4:** Bladder cancer incidence during follow-up in the entire cohort by score interval.

Score Interval	Total Number of Patients, *n*	Number of Patients Who Developed Bladder Cancer During Follow-Up, *n* (%)	
0 to 9	27,211	2	(0.01%)
10 to 19	9094	2	(0.02%)
20 to 29	19,639	5	(0.03%)
30 to 39	45,798	15	(0.03%)
40 to 49	72,284	34	(0.05%)
50 to 59	72,993	90	(0.12%)
60 to 69	77,467	191	(0.25%)
70 to 79	30,761	103	(0.33%)
80 to 89	15,136	105	(0.69%)
90 to 99	12,387	97	(0.78%)

## Data Availability

Data is not available for sharing due to access restriction.

## Supplementary Materials

The following supporting information can be downloaded at: https://www.mdpi.com/article/10.3390/jcm14010004/s1, Table S1: Bladder cancer incidence during follow-up on test set by score interval.
