# Supplementary material for: Point-Based Prediction Model for Bladder Cancer Risk in Diabetes: A Random Survival Forest-Guided Approach
Source: J Clin Med. 2024 Dec 24;14(1):4. doi: 10.3390/jcm14010004 (PMC11720987; doi:10.3390/jcm14010004)
Supplement: Supplementary file 1 [file jcm-14-00004-s001.zip › jcm-3339609-supplementary.pdf]

Table S1. Bladder cancer incidence during follow-up on test set by score interval.

Score interval	Total number of patients, n	Number of patients who developed bladder cancer during follow-up, n (%)
0 to 9	276	0 (0%)
10 to 19	74	0 (0%)
20 to 29	197	2 (1.02%)
30 to 39	472	3 (0.64%)
40 to 49	765	5 (0.65%)
50 to 59	755	19 (2.52%)
60 to 69	841	32 (3.80%)
70 to 79	292	21 (7.19%)
80 to 89	182	14 (7.69%)
90 to 99	138	28 (20.29%)
